# Artificial intelligence for diagnosing microvessels of precancerous lesions and superficial esophageal squamous cell carcinomas: a multicenter study

**DOI:** 10.1007/s00464-022-09353-0

**Published:** 2022-06-15

**Authors:** Xiang-Lei Yuan, Wei Liu, Yan Liu, Xian-Hui Zeng, Yi Mou, Chun-Cheng Wu, Lian-Song Ye, Yu-Hang Zhang, Long He, Jing Feng, Wan-Hong Zhang, Jun Wang, Xin Chen, Yan-Xing Hu, Kai-Hua Zhang, Bing Hu

**Affiliations:** 1 Department of Gastroenterology, West China Hospital, Sichuan University, No. 37 Guo Xue Alley, Wu Hou District, Chengdu, 610041 China; 2 School of Automation, Nanjing University of Information Science and Technology, Nanjing, China; 3 Department of Gastroenterology, Zhongshan Hospital, Xiamen University, Xiamen, China; 4 Department of Gastroenterology, Cangxi People’s Hospital, Guangyuan, China; 5 Department of Gastroenterology, Affiliated Hospital of North Sichuan Medical College, Nanchong, China; 6 The First People’s Hospital of Shuangliu District, Chengdu, China; 7 Xiamen Innovision Medical Technology Co, Ltd., Xiamen, China; 8 ERCDF, Ministry of Education and School of Computing and Software, Nanjing University of Information Science and Technology, Nanjing, China

**Keywords:** Artificial intelligence, Esophageal squamous cell carcinoma, Intrapapillary capillary loops

## Abstract

**Background:**

Intrapapillary capillary loop (IPCL) is an important factor for predicting invasion depth of esophageal squamous cell carcinoma (ESCC). The invasion depth is closely related to the selection of treatment strategy. However, diagnosis of IPCLs is complicated and subject to interobserver variability. This study aimed to develop an artificial intelligence (AI) system to predict IPCLs subtypes of precancerous lesions and superficial ESCC.

**Methods:**

Images of magnifying endoscopy with narrow band imaging from three hospitals were collected retrospectively. IPCLs subtypes were annotated on images by expert endoscopists according to Japanese Endoscopic Society classification. The performance of the AI system was evaluated using internal and external validation datasets (IVD and EVD) and compared with that of the 11 endoscopists.

**Results:**

A total of 7094 images from 685 patients were used to train and validate the AI system. The combined accuracy of the AI system for diagnosing IPCLs subtypes in IVD and EVD was 91.3% and 89.8%, respectively. The AI system achieved better performance than endoscopists in predicting IPCLs subtypes and invasion depth. The ability of junior endoscopists to diagnose IPCLs subtypes (combined accuracy: 84.7% vs 78.2%, *P* < 0.0001) and invasion depth (combined accuracy: 74.4% vs 67.9%, *P* < 0.0001) were significantly improved with AI system assistance. Although there was no significant differences, the performance of senior endoscopists was slightly elevated.

**Conclusions:**

The proposed AI system could improve the diagnostic ability of endoscopists to predict IPCLs classification of precancerous lesions and superficial ESCC.

**Supplementary Information:**

The online version contains supplementary material available at 10.1007/s00464-022-09353-0.

Esophageal cancer is among the most prevalent malignant tumors worldwide and the sixth leading cause of cancer-related death [1]. Esophageal squamous cell carcinoma (ESCC) is the main histological subtype [2], and usually diagnosed at an advanced stage in most patients. The main treatment for advanced ESCC, esophagectomy, is associated with significant mortality [3]. However, if ESCC is diagnosed at an early stage, endoscopic resection (ER) can be performed with an excellent prognosis [4]. According to Japanese [5] and European [6] guidelines, epithelium (EP) or lamina propria (LPM) lesions are definitive indications for ER, and muscularis mucosa (MM) or slight infiltration of the submucosa (< 200 μm; SM1) lesions are relative indications for ER. Esophagectomy or chemoradiotherapy is recommended for treating lesions that more deeply infiltrate the submucosa (≥ 200 μm; SM2 or deeper) lesions. Therefore, precise preoperative assessment of invasion depth is crucial for determining the optimal treatment strategy.

Because the morphologic changes in the intrapapillary capillary loops (IPCLs) pattern correlate with the cancer invasion depth, magnifying endoscopy with narrow band imaging (ME-NBI) plays a critical role in predicting invasion depth of ESCC. Several classification systems for IPCLs morphology have been proposed. The Japanese Endoscopic Society (JES) classification has become widely used in clinical practice because of its simplicity and relatively high accuracy [7]. In this classification system, Type A vessels correspond with normal mucosa or low-grade intraepithelial neoplasia; Type B1, B2 and B3 vessels correspond with invasion into high-grade intraepithelial neoplasia or LPM, MM or SM1, and SM2 or deeper, respectively [7, 8]. However, the IPCLs classification is highly dependent on the experience of endoscopists and subject to interobserver variability. Accurate diagnosis of IPCLs classification will improve the diagnostic accuracy of ESCC invasion depth. Therefore, more advanced methods of mitigating both the complexity and variability associated with IPCLs classification are needed.

Artificial intelligence (AI) using a deep convolutional neural network (DCNN) has developed rapidly in image recognition in various medical fields. Although various studies of the ability of AI systems to detect ESCC or predict its invasion depth have been published [9–13]; however, to our knowledge, few reports on AI systems focus on diagnosing IPCLs classification [14, 15]. Moreover, the reliability of previously reported AI systems that can aid in diagnosing IPCLs subtypes remains questionable owing to the small training and validation datasets and the incomplete applicability (particularly the Type A and Type B3 vessels). This study aimed to develop an AI system based on DCNN to predict the IPCLs subtypes of precancerous lesions and superficial ESCCs, and to explore its role in assisting the endoscopists diagnosis.

## Methods

### Preparation of the training and validation datasets

This retrospective multicenter study was conducted in three hospitals: West China Hospital, Sichuan University (WCHSCU); Nanchong Central Hospital; and Cangxi People’s Hospital. Consecutive patients who underwent ME-NBI examinations for precancerous lesions or superficial ESCC confirmed by histology of endoscopically or surgically resected specimens between January 2014 and April 2021 were included. Patients with a history of esophageal radiotherapy and/or chemotherapy, or with low-quality ME-NBI images (resulting from blur, defocusing, or bleeding) of lesions were excluded. Endoscopic images of eligible patients were collected and the NBI images were selected for the construction of the training and validation datasets. Non-magnified or insufficiently magnified images, low-quality images, and duplicate images were excluded. These images were captured using Olympus equipment (GIF-H260Z, EVIS LUCERA CV-260/CLV-260 (SL), Olympus Medical Systems, Tokyo, Japan) and saved in JPEG format. The structure enhancement was set to B-model level 8.

Three expert endoscopists assessed the quality of all images. Two expert endoscopists (C.C.W and L.J.G), who had a minimum of 10 years of endoscopy experience and had performed more than 300 ME-NBI examinations per year, manually marked the region of representative specific IPCLs subtypes (Type A, B1, B2, or B3 vessels) within each image according to the JES classification (Fig. S1). Avascular areas (AVA) or unclassifiable vessels were not considered in this study in order to simplify the evaluation. Another expert endoscopist (B.H) with a > 20 years of endoscopy experience (> 500 ME-NBI examinations per year) reconfirmed all of the annotated images. Disagreements in the diagnosis were resolved through discussion until the three expert endoscopists reached perfect agreement on the annotation of each image (kappa statistic: 0.809). These annotations were used as the gold standard.

Images taken at WCHSCU between January 2014 and June 2020 were used as the training dataset, while those obtained between July 2020 and April 2021 were used as the internal validation dataset (IVD), which was never used for training. Images obtained from the other two hospitals between November 2019 and November 2020 were used as the external validation dataset (EVD). Images of patients with a single lesion who underwent ER were selected from the IVD and EVD as the ER validation dataset. Due to the ME-NBI images of multiple lesions not corresponding to lesions one to one, patients with multiple lesions were not included in the ER validation dataset. Surgically resected lesions were also not included in the ER validation dataset because the surgical and ER specimens had different cutting intervals (surgical specimen 5 mm; ER specimen 2 mm [15]) and the histological examination of thickly sliced specimens may result in underestimation of the invasion depth.

This study was approved by the Ethics Committee on Biomedical Research, WCHSCU. Informed consent was waived because of the retrospective nature of this study and anonymous data.

### Construction and validation of the AI system

**Fig. 1:**
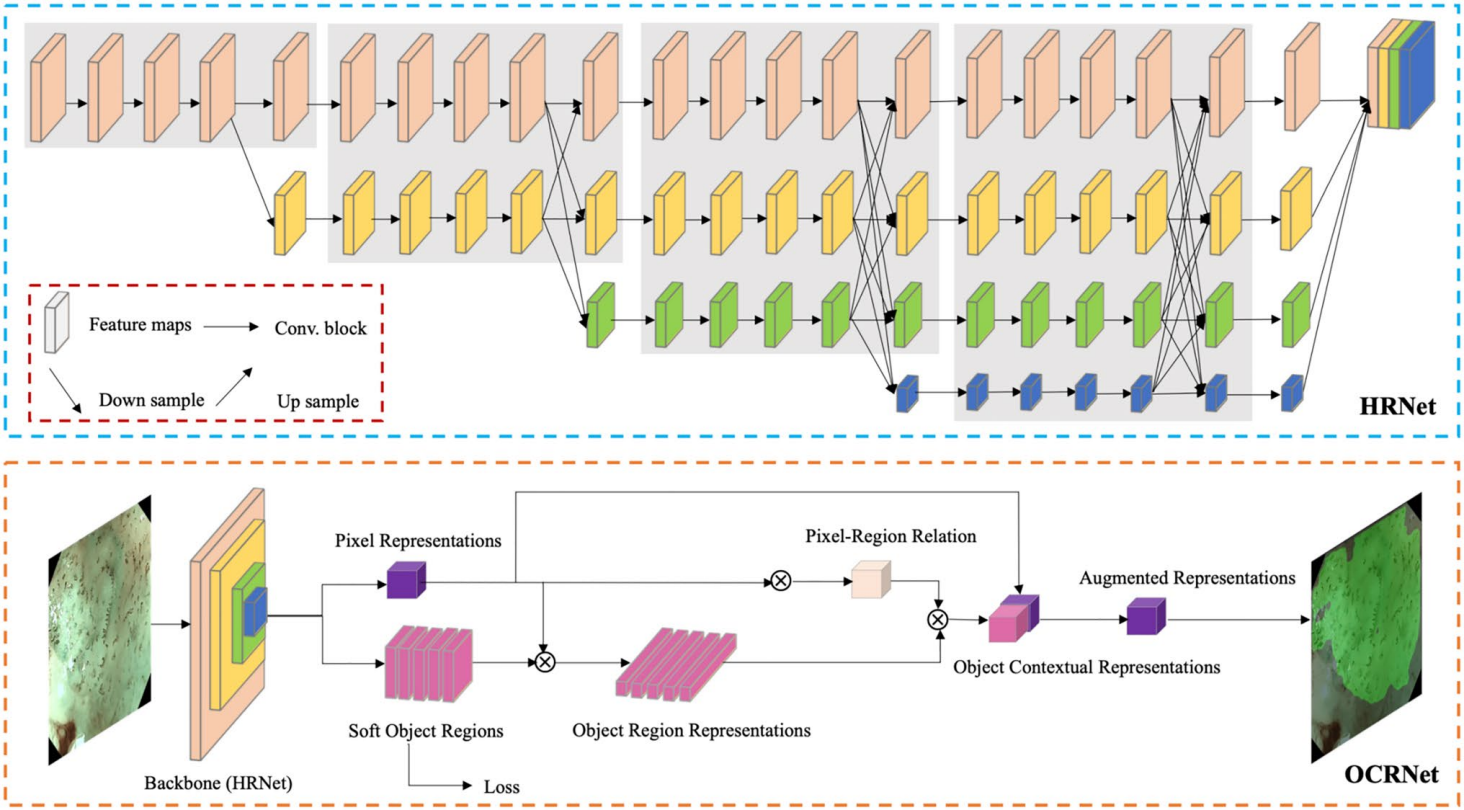
The architecture of the artificial intelligence system [16, 17]

**Fig. 2:**
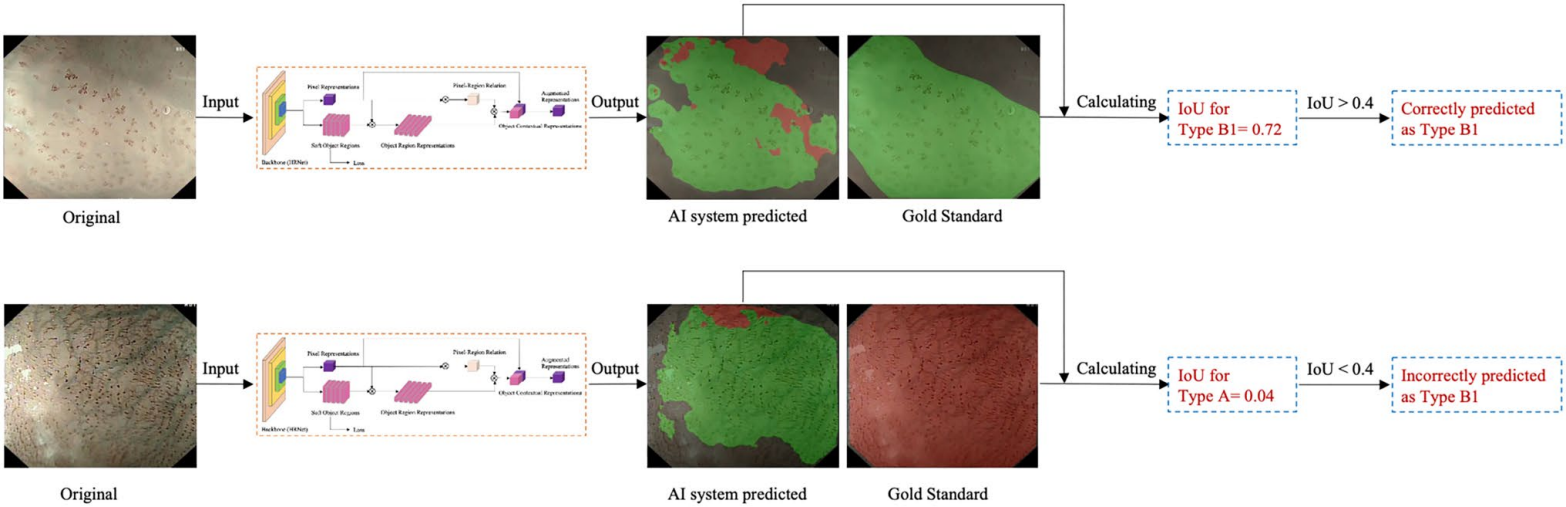
Examples of calculation process of diagnosing intrapapillary capillary loops subtypes by the artificial intelligence system. Red indicates Type A vessels, green indicates Type B1 vessels (Color figure online)

A DCNN algorithm called HRNet+OCR (Fig. 1) was used to train the AI system [16, 17]. The essence of this task is semantic segmentation, namely, the regions of typical IPCLs subtypes on an ME-NBI image are presented by assigning one typical IPCLs label to each pixel of the image. A detailed description of the construction of the AI system is shown in Data S1 and Table S1. When the AI system detected typical IPCLs subtypes, these regions were masked with different colors on the image. Red, green, yellow, and purple indicate Type A, B1, B2, and B3 vessels, respectively. The intersection over union (IoU) between the region of the worst-IPCLs subtype predicted by the AI system and the region of the worst-IPCLs subtype annotated by the endoscopists was calculated. A value of IoU > 0.4 was considered as a correct diagnosis (Fig. 2).

The efficacy of the AI system at diagnosing IPCLs classification was evaluated using the IVD. The robustness of the AI system was assessed using the EVD.

### Comparison between the AI system and endoscopists

Based on the mapping correspondence between the IPCLs classification and the final pathology, the performances of the AI system for predicting IPCLs subtypes of each image and the invasion depth of each lesion were compared with those of endoscopists using the ER validation dataset. The correct diagnosis of invasion depth was confirmed if the prediction of the worst-IPCLs subtype from all the validation images of the lesion was identical to the final pathologic diagnosis of the same lesion. The validity of the gold standard was verified using this dataset.

Eleven endoscopists with various levels of experience from the three hospitals were divided into the senior and junior groups. Four endoscopists with more than 200 ME-NBI examinations per year (endoscopy experience > 8 years) were classified as the senior group, while the other seven endoscopists (endoscopy experience ranged from 2 to 5 years) with at least 6 months training of ME-NBI examinations were classified as the junior group. None of the endoscopists were involved in the selection and annotation of datasets and all were blinded to the clinical information of patients and pathological diagnosis. They were asked to review the ER validation dataset, and independently classify the worst-IPCLs subtype in each image into Type A, Type B1, Type B2, or Type B3 vessels.

To explore the assistance ability of the AI system, all endoscopists were required to make a diagnosis again on the same dataset after 1 month, referring to the results given by the AI system. The performance of the endoscopists with and without AI system assistance was then compared.

The acceptance of AI system assistance by the endoscopists is an important factor in clinical practice. This factor can be reflected by the individual personality traits, which are assessed using a grit scale [18, 19]. The grit scale includes two components (consistency of interest and perseverance of effort) and is validated based on a questionnaire containing 12 items. Each item is scored on a 5-point scale (from 1 to 5). The summed score is divided by 12 to obtain the final score.

### Outcome measures and statistical analysis

The accuracy, sensitivity, specificity, and diagnostic time were calculated. Detailed definitions of these values are shown in Data S1. The results of the endoscopists were presented as averages. A two-sided McNemar test was used to compare the different groups. The interobserver agreement of the endoscopists was calculated using Fleiss’ kappa statistics. Grit scores were analyzed using correlation and linear regression analysis. Two-sided statistical tests were performed, and statistical significance was set at *P* < 0.05. The statistical analysis was performed using SPSS (version 26.0; IBM Corp., Armonk, NY, USA).

## Results

### Characteristics of patients and lesions in the datasets

**Fig. 3:**
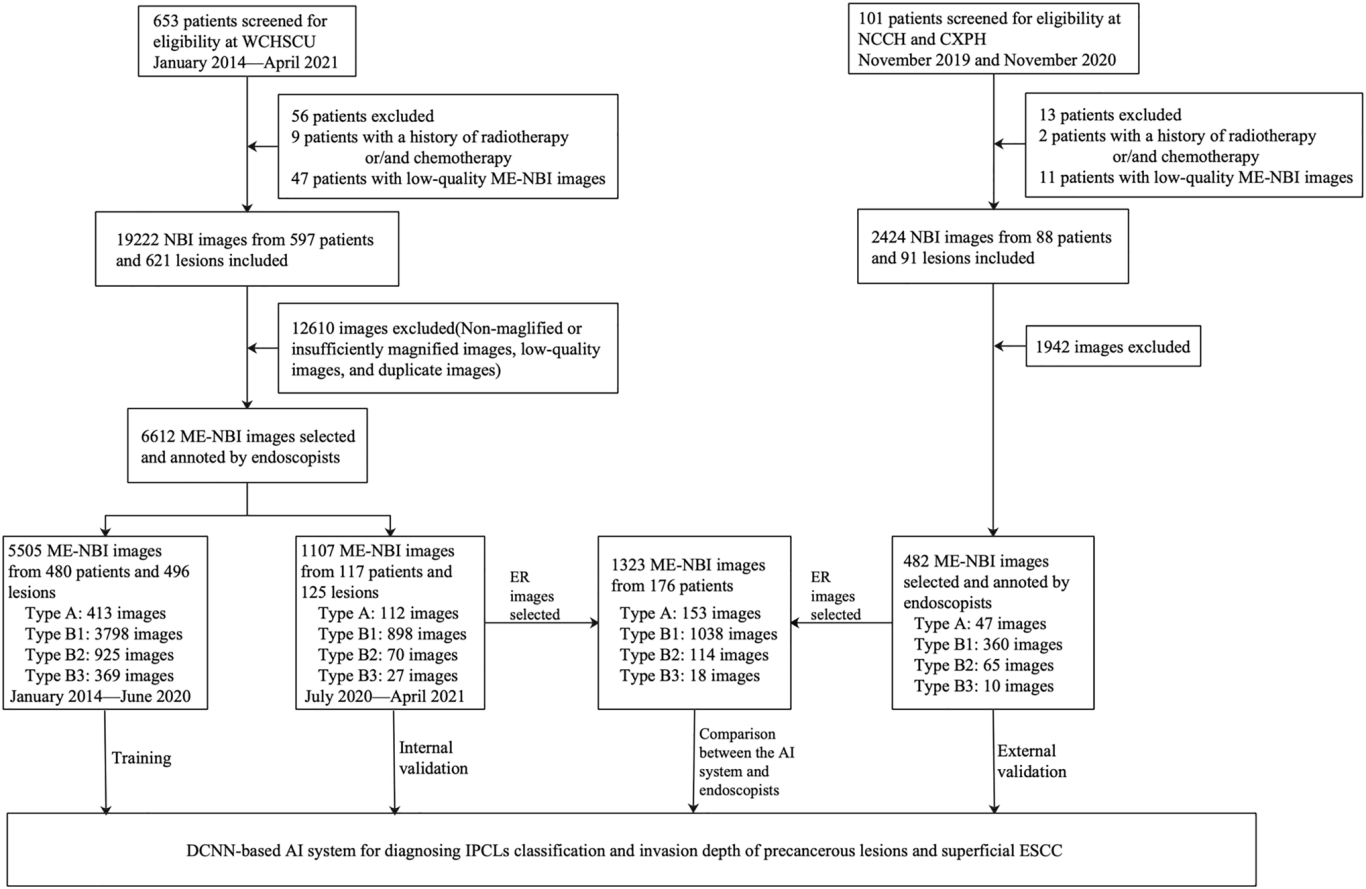
Flowchart of selection of patients and images

A flowchart of the patients and images selection process is shown in Fig. 3. After selection and annotation, 7094 images from 685 patients and 712 lesions were used to train and validate the AI system. The detailed clinicopathological characteristics of the selected patients and lesions in the training and validation datasets are summarized in Table 1.

**Table 1:** Characteristics of patients and lesions in the datasets

	Training dataset	Internal validation dataset	External validation dataset	ER validation dataset
Study period	Jan 2014–Jun 2020	Jul 2020–Apr 2021	Nov 2019–Nov 2020	Nov 2019–Apr 2021
Patient characteristics	480	117	88	176
Sex, male/female	345/135	89/28	48/40	113/63
Age (year), median (range)	62 (30–83)	64 (48–83)	66 (35–79)	65 (35–83)
Lesion characteristics	496	125	91	176
Location (upper/middle/lower)	87/235/174	25/62/38	20/50/21	34/92/50
Size (mm), median (range)	25 (3–100)	25 (3–90)	29 (5–70)	22 (3–80)
Macroscopic type, 0–I/0–IIa/0–IIb/0–IIc/0–IIa + IIc	18/108/241/78/51	5/27/72/10/11	2/15/53/14/7	0/31/110/21/14
Invasion depth				
Endoscopic resection	416	112	85	176
LGIN/HGIN/LPM/MM/SM1/SM2 or deeper	57/161/98/48/14/38	11/45/35/12/4/5	7/52/7/12/3/4	16/84/38/24/5/9
Surgical resection	80	13	6	–
HGIN/LPM/MM/SM	10/2/9/59	1/4/0/8	0/0/0/6	–
Annotated images	5505	1107	482	1323
Type A	413	112	47	153
Type B1	3798	898	360	1038
Type B2	925	70	65	114
Type B3	369	27	10	18

*ER* endoscopic resection; *LGIN* low-grade intraepithelial neoplasia; *HGIN* high-grade intraepithelial neoplasia; *LPM* lamina propria; *MM* muscularis mucosa; *SM1* slight infiltration of the submucosa (< 200 μm); *SM2* deeper infiltration of the submucosa($\ge$ 200 μm)

### Performance of the AI system in internal and external validation datasets

**Fig. 4:**
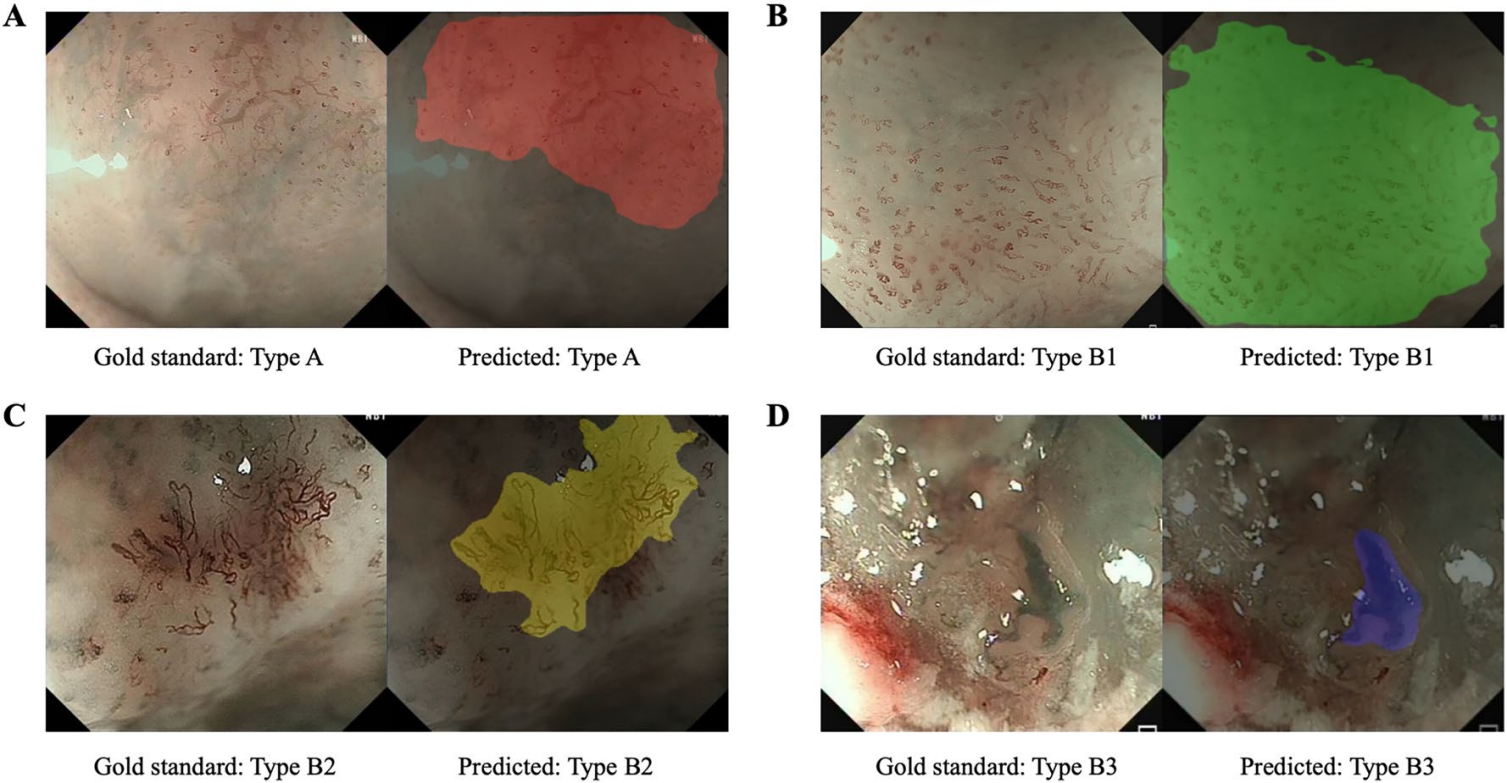
Representative images diagnosed by the artificial intelligence system. (A) Type A, (B) Type B1, (C) Type B2, (D) Type B3

The AI system accurately diagnosed IPCLs subtypes in the IVD and EVD (Fig. S2, Table S2). The combined diagnostic accuracies were 91.3% for the IVD and 89.8% for the EVD. In the IVD, the individual sensitivities were 92.9% for Type A, 91.9% for Type B1, 85.7% for Type B2, and 81.5% for Type B3. Satisfactory individual sensitivities were also obtained in the EVD (97.9% for Type A, 89.2% for Type B1, 89.2% for Type B2, and 80.0% for Type B3). Misdiagnoses mainly involved Type B2 and B3 vessels. Type B2 was often mistaken for Type B1, while Type B3 was often mistaken for Type B2. The detailed accuracy, sensitivity, and specificity of the AI system in the IVD and EVD are shown in Table 2. Examples of AI system-diagnosed images are shown in Fig. 4.

**Table 2:** Performance of the AI system for diagnosis of IPCLs classification in the IVD and EVD

	Accuracy	Sensitivity	Specificity
IVD			
Total	91.3 (89.7–93.0)	–	–
Type A	93.7 (92.2–95.1)	92.9 (88.0–97.7)	93.8 (92.3–95.3)
Type B1	91.8 (90.2–93.4)	91.9 (90.1–93.7)	91.4 (87.6–95.2)
Type B2	97.7 (96.9–98.6)	85.7 (77.3–94.1)	98.6 (97.8–99.3)
Type B3	99.5 (99.0–99.9)	81.5 (65.8–97.1)	99.9 (99.7–100)
EVD			
Total	89.8 (87.1–92.5)	–	–
Type A	91.7 (89.2–94.2)	97.9 (93.6–100)	91.0 (88.3–93.7)
Type B1	90.5 (87.8–93.1)	89.2 (85.9–92.4)	94.3 (90.1–98.4)
Type B2	98.2 (97.0–99.4)	89.2 (81.5–97.0)	99.5 (98.9–100)
Type B3	99.4 (98.7–100)	80.0 (49.8–100)	99.8 (99.4–100)

The data were present as value% (95% confidence interval)

*AI* artificial intelligence; *IPCLs* intrapapillary capillary loops; *IVD* internal validation dataset; *EVD* external validation dataset

The AI system could process 23 frames per second on a high-speed computer, which can be adapted as real-time diagnosis. We successfully tested the AI system in real ME-NBI videos. As shown in Video S1, the AI system accurately diagnosed the worst-IPCLs subtypes.

### Comparison between the AI system and endoscopists in ER validation dataset

The senior and junior endoscopists required about 3.2 s and 6.9 s to diagnose an image, respectively, whereas the AI system required only 0.04 s per image. In the ER validation dataset, the AI system also exhibited good performance at diagnosing IPCLs subtypes, with a combined accuracy of 91.4%. Although senior endoscopists showed better diagnostic ability than junior endoscopists, their combined accuracies were significantly lower than that of the AI system (senior: 87.1%, *P* < 0.0001; junior: 78.2%, *P* < 0.0001) (Tables 3, S3, Fig. S3). Regarding the individual accuracies, sensitivities and specificities of IPCLs subtypes, except for Type B3, the performance of the AI system for diagnosing the other three IPCLs subtypes was significantly better than that of senior and junior endoscopists. The interobserver agreement among senior endoscopists was moderate (*k*: = 0.596), while it was fair among junior endoscopists (*k* = 0.397) (Table 5). Misdiagnoses by endoscopists were mainly involved in Type A, B2 and B3. Type A and B2 were often mistaken for Type B1, while Type B3 was often mistaken for Type B2.

**Table 3:** Performance of the AI system and endoscopists for diagnosis of IPCLs classification in the ER validation dataset

Types	Groups	Non-assisted			AI-assisted		
		Accuracy	Sensitivity	Specificity	Accuracy	Sensitivity	Specificity
Total	AI system	91.4(89.9–92.9)	–	–	–	–	–
	Senior	87.1 (86.2–88.0)^†^	–	–	88.3 (87.4–89.1)^†^	–	–
	Junior	78.2 (77.4–79.0)^†^	–	–	84.7 (84.0–85.4)^†‡^	–	–
Type A	AI system	93.2 (91.8–94.6)	94.1 (90.3–97.9)	93.1 (91.6–94.5)	–	–	–
	Senior	94.3 (93.7–95.0)^†^	78.1 (74.8–81.4)^†^	96.5 (95.9–97.0)^†^	94.6 (93.9–95.2)^†^	71.7 (68.2–75.3)^†‡^	97.5 (97.1–98.0)^†‡^
	Junior	90.1 (89.5–90.7)^†^	58.4 (55.4–61.3)^†^	94.3 (93.8–94.8)	91.2 (90.7–91.8)^‡^	76.0 (73.1–79.0)^†‡^	92.8 (92.2–93.3)^‡^
Type B1	AI system	91.6 (90.1–93.1)	91.6 (89.9–93.3)	91.6 (88.3–94.8)	–	–	–
	Senior	88.3 (87.5–89.2)^†^	90.9 (90.0–91.8)	79.0 (76.7–81.4)^†^	89.8 (89.0–90.6)^†‡^	92.8 (92.0–93.6)^†^	78.9 (76.5–81.2)^†^
	Junior	80.4 (79.6–81.2)^†^	82.2 (81.3–83.0)^†^	73.8 (71.9–75.8)^†^	85.5 (84.8–86.3)^†‡^	85.5 (84.6–86.3)^†‡^	85.9 (84.3–87.4)^‡^
Type B2	AI system	98.3 (97.6–99.0)	87.7 (81.6–93.8)	99.3 (98.8–99.7)	–	–	–
	Senior	92.9 (92.2–93.6)^†^	72.6 (68.5–76.7)^†^	94.8 (94.2–95.5)^†^	93.8 (93.2–94.5)^†^	75.9 (71.9–79.8)†	95.5 (94.9–96.1)^†^
	Junior	88.4 (87.7–89.0)^†^	73.2 (70.1–76.3)^†^	89.8 (89.2–90.5)^†^	93.6 (93.1–94.1)^†‡^	79.6 (76.6–82.5)	94.8 (94.3–95.3)^†‡^
Type B3	AI system	99.7 (99.4–100)	77.8 (56.5–99.1)	100 (100–100)	–	–	–
	Senior	98.6 (98.3–99.0)	37.5 (26.0–49.0)	99.5 (99.3–99.7)	98.4 (98.1–98.7)	47.2 (35.4–59.0)	99.1 (98.8–99.4)
	Junior	97.5 (97.2–97.9)	50.0 (41.1–58.9)	98.2 (97.9–98.5)	98.3 (98.1–98.6)	54.3 (45.1–63.5)	98.9 (98.7–99.1)

The data were present as value% (95% confidence interval)

*AI* artificial intelligence; *IPCLs* intrapapillary capillary loops; *ER* endoscopic resection

^†^Significant difference between the AI system and the target group

^‡^Significant difference between the results of groups without AI-assisted and with AI-assisted

With regard to invasion depth, the gold standard exhibited satisfactory diagnostic ability. Although the combined accuracy of the AI system (80.7%) was lower than that of the gold standard (84.7%), this value was significantly higher than those of the senior (73.9%, *P* < 0.0001) and junior (67.9%, *P* < 0.0001) endoscopists (Table 4, Fig. S4). For EP-LPM lesions and MM-SM1 lesions, the individual accuracy, sensitivity and specificity of the AI system were better than those of both endoscopists subgroups. The AI system showed comparable performance in diagnosing SM2-deeper lesions to both endoscopists subgroups. The interobserver agreement among both endoscopists subgroups was moderate (Senior: *k* = 0.573; junior: *k* = 0.488) (Table 5).

**Table 4:** Performance of the AI system and endoscopists for prediction of invasion depth in the ER validation dataset

Types	Groups	Non-assisted			AI-assisted		
		Accuracy	Sensitivity	Specificity	Accuracy	Sensitivity	Specificity
Total	Gold standard	84.7 (79.3–90.0)	–	–	–	–	–
	AI system	80.7 (74.8–86.6)	–	–	–	–	–
	Senior	73.9 (70.6–77.1)^†^	–	–	77.4 (74.3–80.5)^†^	–	–
	Junior	67.9 (65.2–70.5)^†^	–	–	74.4 (72.0–76.9)^†‡^	–	–
EP-LPM	Gold standard	87.5 (82.6–92.4)	92.8 (99.4–97.1)	68.4 (52.9–83.9)	–	–	–
	AI system	83.0 (77.3–88.6)	90.6 (85.6–95.5)	55.3 (38.7–71.8)	–	–	–
	Senior	77.7 (74.6–80.8)^†^	82.2 (79.0–85.4)^†^	61.2 (53.3–69.0)	80.1 (77.2–83.1)^†^	86.1 (83.2–88.9)^†^	58.6 (50.6–66.5)
	Junior	71.5 (69.0–74.0)^†^	72.0 (69.2–74.9)^†^	69.5 (64.0–75.1)	77.5 (75.2–79.9)^†‡^	80.8 (78.4–83.3)^†‡^	65.4 (59.7–71.2)
MM-SM1	Gold standard	85.8 (80.6–91.0)	58.6 (39.6–77.7)	91.2 (86.5–95.8)	–	–	–
	AI system	83.5 (78.0–89.1)	51.7 (32.4–71.1)	89.8 (84.8–94.7)	–	–	–
	Senior	76.1 (73.0–79.3)^†^	50.9 (41.6–60.1)	81.1 (78.0–84.3)^†^	80.4 (77.5–83.3)^†^	53.4 (44.2–62.7)	85.7 (82.9–88.6)^†^
	Junior	72.1 (69.6–74.6)^†^	57.6 (50.8–64.5)	74.9 (72.3–77.6)^†^	78.2 (75.9–80.6)^†‡^	57.6 (50.8–64.5)	82.3 (80.0–84.6)^†‡^
SM2-deeper	Gold standard	96.0 (93.1–98.9)	44.4 (3.9–85.0)	98.8 (97.1–100)	–	–	–
	AI system	94.9 (91.6–98.2)	22.2 (5.0–56.1)	98.8 (97.1–100)	–	–	–
	Senior	93.9 (92.1–95.7)	19.4 (5.9–33.0)	97.9 (96.8–99.0)	94.3 (92.6–96.0)	22.2 (8.0–36.5)	98.2 (97.2–99.2)
	Junior	92.1 (90.6–93.6)	28.8 (18.6–38.9)	96.5 (95.5–97.6)	93.1 (91.7–94.5)	19.0 (9.1–29.0)	96.5 (95.4–97.5)

The data were present as value% (95% confidence interval)

*AI* artificial intelligence; *ER* endoscopic resection; *EP* epithelium; *LPM* lamina propria mucosa; *MM* muscularis mucosa; *SM1* slight infiltration of the submucosa (< 200 μm); *SM2* deeper infiltration of the submucosa($\ge$ 200 μm)

^†^Significant difference between the AI system and the target group

^‡^Significant difference between the results of groups without AI-assisted and with AI-assisted

**Table 5:** The interobserver agreement among endoscopists

	IPCLs classification diagnosis		Invasion depth prediction	
	Non-assisted	AI-assisted	Non-assisted	AI-assisted
Senior	0.596 (0.596–0.597)	0.607 (0.606–0.607)	0.573 (0.571–0.574)	0.593 (0.591–0.595)
Junior	0.397 (0.397–0.398)	0.691 (0.691–0.691)	0.488 (0.487–0.489)	0.694 (0.693–0.695)

The data were present as value% (95% confidence interval)

*IPCLs* intrapapillary capillary loops; *AI* artificial intelligence

### Assistant efficiency of the AI system for endoscopists

**Fig. 5:**
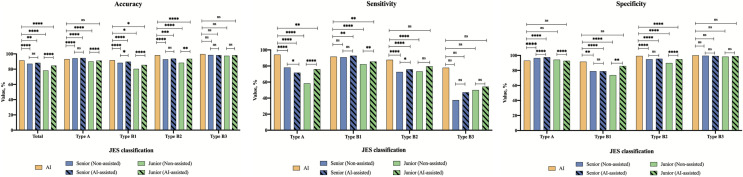
Comparison between the AI system and endoscopists in the diagnosis of IPCLs subtypes. *AI* artificial intelligence; *IPCLs* intrapapillary capillary loops

With AI system assistance, the diagnostic times of the senior (2.6 vs 3.2 s per image) and junior (5.8 vs 6.9 s per image) endoscopists were both slightly reduced. The combined accuracy of the senior endoscopists for diagnosing IPCLs subtypes was slightly improved (88.3% vs 87.1%, *P* = 0.071), while that of the junior endoscopists was significantly increased (84.7% vs 78.2%, *P* < 0.0001) (Tables 3, S3, Figs. 5, S5). The individual accuracy, sensitivity, and specificity of the junior endoscopists for diagnosing Type A, B1, and B2 were remarkably increased. For Type B3, although there was no statistical difference, the diagnostic performance of endoscopists assisted by the AI system was also slightly improved.

**Fig. 6:**
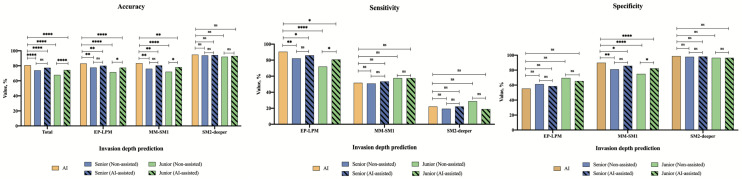
Comparison between the AI system and endoscopists in the prediction of invasion depth

The performance of junior endoscopists at predicting invasion depth was remarkably improved with AI system assistance, especially for EP-LPM lesions and MM-SM1 lesions (Tables 4, S3, Figs. 6, S6). The interobserver agreement for IPCLs subtypes diagnosis and invasion depth prediction among both endoscopist subgroups was improved with AI system assistance (Table 5).

### Personality traits and acceptance of AI system assistance

As shown in Table 6, there was no significant correlation between personality traits and diagnostic accuracy with or without AI assistance.

**Table 6:** Strength of correlation between grit score and diagnostic accuracy of endoscopists

	Score		IPCLs classification diagnosis				Invasion depth prediction			
			Non-assisted		AI-assisted		Non-assisted		AI-assisted	
	Mean ± SD	Range	Correlation, *r*	*P*	Correlation, *r*	*P*	Correlation, *r*	*P*	Correlation, *r*	*P*
Grit score	3.64 ± 0.59	2.42–4.42	0.180	0.596	0.135	0.693	0.129	0.706	0.231	0.495
Consistency of interest	3.71 ± 0.68	2.17–4.67	0.193	0.569	0.240	0.477	0.428	0.189	0.094	0.784
Perseverance of effort	3.58 ± 0.91	2.17–4.83	0.090	0.792	0.005	0.989	0.154	0.652	0.371	0.261

*IPCLs* intrapapillary capillary loops; *AI* artificial intelligence; *SD* standard deviation

## Discussion

In this study, we developed an AI system to assist endoscopists with IPCLs classification of precancerous lesions and superficial ESCCs. To the best of our knowledge, our AI system was the first diagnostic system covering almost all IPCLs classification (Type A, B1, B2, and B3 vessels) on precancerous lesions and superficial ESCCs. This was also the first study to evaluate the assistant role of the AI system for diagnosing IPCLs subtypes by endoscopists using multicenter validation datasets.

Previous studies have reported the use of DCNN-based AI systems to diagnose IPCLs subtypes or ESCC invasion depth. Zhao et al. [14] have developed an AI system to automatically classify IPCLs subtypes. The overall accuracy of the AI system in diagnosing Type A, B1, and B2 vessels was significantly higher than that of junior endoscopists, but this study excluded Type B3 vessels due to the low numbers of images. The presence of Type B3 vessels is of great significance in the diagnosis of invasion depth of ESCCs, because it suggests that deeper invasion than SM2, indicating the need for esophagectomy or chemoradiotherapy. Although the AI system reported by Uema et al. [15] could diagnose Type B3 vessels, and the overall accuracy of IPCLs classification of the AI system was remarkably higher than that of experts. Not only *did* the datasets of this study not include Type A vessels, but also the datasets were composed of images of typical IPCLs subtypes cropped from the original images, which weakened the clinical practicability of the AI system. Everson et al. [8, 20] have successively reported AI systems for classifying IPCLs patterns as normal (Type A) or abnormal (Type B). However, these AI systems could not classify the subtypes of Type B vessels, so they were limited in diagnosing invasion depth. Tada and his colleagues [11–13] have successively reported the use of multiple AI systems to distinguish EP-SM1 lesions from SM2 or deeper lesions on white-light imaging, non-ME-NBI or blue laser imaging. However, an AI system that can directly diagnose the invasion depth should be of more clinical significance. In addition, the validation datasets of previous studies were relatively small, and they were all conducted in a single center, lacking the verification of robustness of the AI system. The present study used complete images of IPCLs subtypes to develop the AI system as well as datasets from three different hospitals to verify the diagnostic robustness of the AI system. Moreover, we compared the performances of the AI system and endoscopists and evaluated the assist role of the AI system in the diagnostic performance of endoscopists.

Our AI system showed excellent performance in the IVD and EVD, indicating its tremendously generalized ability. The performance of the AI system was obviously better than that of the endoscopists, showing the potential ability to assist endoscopists with diagnosis. This conjecture was confirmed by comparing the diagnostic results of endoscopists with and without AI system assistance. Additionally, the diagnostic times of both endoscopist subgroups assisted by the AI system were slightly shortened, indicating that the AI system may help to increase the diagnostic efficiency. Moreover, the interobserver agreement of junior endoscopists was comparable to that of senior endoscopists with AI system assistance. This indicated that the AI system could reduce the diagnostic discrepancies and promote the homogenization of diagnostic performance. The acceptance of AI systems by endoscopists is crucial. A previous study [21] showed that higher grit correlated with the flexible acceptance of AI system assistance. However, there was no significant correlation between grit scores and diagnostic accuracy with or without AI system assistance in the current study, suggesting that the personality traits of endoscopists did not affect acceptance of the AI system.

We found that the sensitivity of the AI system or endoscopists for Type B2 and Type B3 vessels tended to be lower than that of other vessel types. This may be due to the wide range of interpretation of Type B2 vessels and the incorrect diagnosis of Type B2 or branching vessels as Type B3 vessels by the AI system. Moreover, the rate of Type B3 vessels was low [22], and the limited number of images may have affected the results. Type A vessels are often confused with Type B1 vessels by some endoscopists. The sensitivities of junior endoscopists for Type A and Type B1 vessels were both significantly improved with AI system assistance. Although the sensitivity of senior endoscopists for Type B1 vessels increased slightly after referring to the results of the AI system, their sensitivity for Type A vessels decreased significantly because senior endoscopists probably tended to insist on their *initial* judgement when their diagnoses were inconsistent with those of the AI system.

Although the morphology of microvessels on the surface of ESCC is related to the cancer invasion depth, several studies [7, 22, 23] have shown that the use of the JES classification does indeed over- or underestimate the invasion depth. Therefore, the difference between the gold standard and the final pathological diagnosis for predicting invasion depth in the current study is understood. The overall accuracy of the JES classification at predicting ESCC invasion depth is reportedly 78.6–90.5% [7, 24]. The overall accuracies of the gold standard (84.7%) and AI system (80.7%) in the current study were similar to those reported in previous studies.

Nevertheless, this study had some limitations. First, there were more images of Type B1vessels than those of the other three IPCLs subtypes. Unbalanced data may lead to a low diagnostic sensitivity of the AI system to some IPCLs subtypes. When training the AI system, we used data enhancement to expand the images with less data, and used weighted loss function to solve the problem of substantially unbalanced data. We will further collect more images and videos to optimize the AI system. Second, no images of AVA vessels were included in this study, which may have affected the accurate mapping correspondence between IPCLs classification and invasion depth. We collected these images to optimize the AI system. Third, the AI system was developed using high-quality images. The satisfactory performance of the AI system may not reflect its ability in real-world situations. Fourth, selection bias could not be avoided in this retrospective study.

In conclusion, here we developed an AI system that could aid endoscopists in predicting IPCLs subtypes of precancerous lesions and superficial ESCC. However, further studies are required to optimize the AI system and evaluate its efficacy.

## Supplementary Information

Below is the link to the electronic supplementary material.

Supplementary file1 (DOCX 3006 kb)

Supplementary file2 (MP4 79311 kb)—**Video S1** The AI system diagnosed the IPCLs subtypes by covering the corresponding regions with different colors. Red, green, yellow and purple indicate Type A, B1, B2, and B3 vessels, respectively.
