# Supplementary material for: Artificial intelligence for diagnosing microvessels of precancerous lesions and superficial esophageal squamous cell carcinomas: a multicenter study
Source: Surg Endosc. 2022 Jun 15;36(11):8651–62. doi: 10.1007/s00464-022-09353-0 (PMC9613556; doi:10.1007/s00464-022-09353-0)
Supplement: Supplementary file 1 — Supplementary file1 (DOCX 3006 kb) [file 464_2022_9353_MOESM1_ESM.docx]

**Supplementary Material**

**Data S1.** **Details of construction of the artificial intelligence system and definitions of outcomes.**

A deep convolutional neural network algorithm called HRNet+OCR was used to train an artificial intelligence (AI) system for predicting intrapapillary capillary loops (IPCLs) classification of precancerous lesions and superficial esophageal squamous cell carcinoma (ESCC). The essence of this task is semantic segmentation, namely, the regions of the typical IPCLs subtypes (Type A, Type B1, Type B2, or Type B3) on the image are presented by assigning one typical IPCLs label to each pixel of the image.

An HRNet (High-Resolution Net) is used as the backbone for extracting features from the images[1], and its final representation is fed into an OCR (Object-Contextual Representation) module to obtain the final segmentation prediction[2]. The HRNet is composed of parallel high-to-low multiresolution subnetworks with multiscale fusions by repeatedly exchanging the information across the multiresolution subnetworks. The HRNet can not only maintain a high resolution throughout the whole process rather than recovering the resolution through a low-to-high process, it can also realize multiscale fusions. Thus, the predicted regions of typical IPCLs subtypes are potentially more precise and accurate. The main idea of the OCR is that the label of a pixel is the category label of the object to which the pixel belongs. The OCR strengthens the pixel representation by characterizing each pixel with the corresponding object region representation to achieve better pixel labeling.

The framework used for implementation was PyTorch. The AI system was trained using an NVIDIA GeForce RTX 2080Ti as a graphics processing unit. A stochastic gradient descent algorithm with a momentum of 0.9 and a weight decay rate of 5 × 10^-4^ was used for system training. The input images were resized to 512 × 512 pixels. The method of resizing the image is as follows: First, the black edge area displaying image information was removed, and only the central endoscopic image was retained. Then, the image was uniformly resized to 512 x 512 using bilinear interpolation. Finally, the adjusted images were input into the network for training. Cross-entropy loss was used as loss function. The learning rate was adjusted using a Poly strategy in which the basic learning rate was 0.01.

For the problem of substantially unbalanced data in the training process, we mainly solve it from two aspects:

(1) Using data enhancement

The data enhancement method was used to expand the images with less data. For example, we extracted the area of Type B3 in the original image by matting, and then randomly pasted it on the images that did not contain Type B3, so as to expand the number of Type B3 images.

(2) Using weighted loss function

Medium frequency balancing method was used to weight the loss of each category in the Cross-entropy loss. That is, the weighted loss of each category during training was calculated by counting the number of images of each category, the number of pixels of each category and the total number of pixels of the image corresponding to each category.

For an image dataset containing multiple categories, each category had a class frequency. The number of pixels of each category was divided by the total number of pixels of the dataset to calculate the median value of all class frequencies, and then divided by the frequency corresponding to the category to obtain the weight:

$$\alpha_{c},\alpha_{c}=\frac{\text{ median\_freq }}{\text{ }\text{freq}\text{(c) }}$$

$$\text{freq}\text{ }(c)=\frac{\text{ sum\_pixel\_c }}{\text{ }\text{sum\_image\_pixel\_c}\text{ }}$$

A weight of each pixel is gave.

$c$ represents the category to which the pixel belongs.

$\text{median\_freq}$ represents the median of the class ferquencies.

$\text{sum\_pixel}\text{\_}c$ represents the sum of all pixels of the category. For example, a dataset of 100 images contains 80 images of Type B1, the number of pixels of Type B1 in these 80 images is calculated.

$\text{sum\_image\_pixel}\text{\_}c\text{ }$represents the sum of the number of pixels of all images.

During the training process, for each input image, the classification and region of a typical IPCLs subtype computed by the AI system were compared with the annotations of the experienced endoscopists. The parameters of the AI system were then modified slightly to decrease the error in the same image. The repeated process was then performed multiple times for each image in the training dataset. During the validation process, when the AI system detected typical IPCLs subtypes from the image in the validation datasets, the regions of these IPCLs subtypes were masked on this image with different colors. Red, green, yellow and purple indicate Type A, B1, B2, and B3 vessels, respectively.

During the calculation process, because only the worst-IPCLs subtype in each image is of concern, the real mask of each annotated validation image is processed, and only the region of the worst-IPCLs subtype is retained, and the newly generated mask is used as the final mask for calculation. The intersection over union (IoU) between the mask of the IPCLs subtype predicted by the AI system and the mask of the worst-IPCLs subtype annotated by the experienced endoscopists was calculated. The value of IoU > 0.4 was considered the correct classification. In terms of the definition of classification labels, the correctly classified label corresponds to the mask of the worst-IPCLs subtype. The label of the incorrect classification is the label corresponding to the mask of the incorrectly predicted IPCLs subtype with the most occurrences of each pixel in the segmentation map.

From our previous experience of developing the AI system for endoscopy[3] and based on a previous study[4], the best cutoff value for cancer ranged from 0.4 to 0.5. To choose an appropriate value, three expert endoscopists involved in image annotation were asked to review the results predicted by the AI system and chose those acceptable results with the IoU value blinded. Through this method, the cutoff value of 0.4 was selected, thereby ensuring high sensitivity.

During the development of the AI system, we verified the performance of the AI system. The training dataset described in this study were randomly assigned to the training set and the validation set for AI system development (8:2). The final AI system showed good performance in diagnosing IPCLs subtypes in the validation set for AI system development (Table S1).

1. Accuracy was defined as the proportion of the number of correctly predicted samples in total number of samples. Accuracy=(TP+TN)/(TP+FP+TN+FN).
2. Sensitivity was defined as the proportion of correctly predicted positive samples in all actual positive samples. Sensitivity=TP/(TP+FN)
3. Specificity was defined as the proportion of correctly predicted negative samples in all actual negative samples. Specificity=TN/(TN+FP)
4. IoU=$\frac{area \left( predicted regions \right)\cap area (ground truth regions)}{area \left( predicted regions \right)\cup area (ground truth regions)}$

TP: true positive; TN: true negative; FP: false positive; FN : false negative;

IoU: intersection over union.

**References**

1. Sun K, Xiao B, Liu D, Wang J (2019) Deep High-Resolution Representation Learning for Human Pose Estimation. Proceedings of the IEEE/CVF Conference on Computer Vision and Pattern Recognition, pp 5693-5703

2. Yuan Y, Chen X, Chen X, Wang J (2019) Segmentation transformer: Object-contextual representations for semantic segmentation. arXiv preprint arXiv:190911065

3. Yuan XL, Guo LJ, Liu W, Zeng XH, Mou Y, Bai S, Pan ZG, Zhang T, Pu WF, Wen C, Wang J, Zhou ZD, Feng J, Hu B (2022) Artificial intelligence for detecting superficial esophageal squamous cell carcinoma under multiple endoscopic imaging modalities: A multicenter study. J Gastroenterol Hepatol 37**:**169-178

4. Ohmori M, Ishihara R, Aoyama K, Nakagawa K, Iwagami H, Matsuura N, Shichijo S, Yamamoto K, Nagaike K, Nakahara M, Inoue T, Aoi K, Okada H, Tada T (2020) Endoscopic detection and differentiation of esophageal lesions using a deep neural network. Gastrointest Endosc 91**:**301-309

**Table S1 Performance of the AI system for diagnosing IPCLs classification in the validation set for AI system development.**

|  | **Number of images** | **Accuracy** | **Sensitivity** | **Specificity** |
| --- | --- | --- | --- | --- |
| **Total** | 1099 | 91.5 (89.9-93.2) | - | - |
| **Type A** | 82 | 95.4 (94.1-96.6) | 93.9 (88.6-99.2) | 95.5 (94.2-96.8) |
| **Type B1** | 759 | 92.5 (91.0-94.1) | 92.8 (90.9-94.6) | 92.1 (89.2-94.9) |
| **Type B2** | 185 | 96.5 (95.4-97.5) | 89.2 (94.7-93.7) | 97.9 (97.0-98.8) |
| **Type B3** | 73 | 98.7 (98.1-99.4) | 82.2 (73.2-91.2) | 99.9 (99.7-100) |

AI, artificial intelligence; IPCLs, intrapapillary capillary loops.

**Table S2 The mIoU of the AI system for diagnosing IPCLs subtypes in different validation datasets.**

|  | **Internal validation dataset** | **External validation dataset** | **ER validation dataset** |
| --- | --- | --- | --- |
| **Type A** | 69.9 | 63.2 | 64.5 |
| **Type B1** | 71.0 | 77.2 | 74.7 |
| **Type B2** | 61.6 | 52.1 | 57.3 |
| **Type B3** | 47.2 | 43.5 | 45.9 |

mIoU, mean intersection over union; AI, artificial intelligence; IPCLs, intrapapillary capillary loops.

The data were present as value%

**Table S3 The *P* values of the comparison tests.**

|  |  | **IPCLs classification diagnosis** | | | | | **Invasion depth prediction** | | | |
| --- | --- | --- | --- | --- | --- | --- | --- | --- | --- | --- |
|  |  | **Total** | **Type A** | **Type B1** | **Type B2** | **Type B3** | **Total** | **EP-LPM** | **MM-SM1** | **SM2-deeper** |
| **Accuracy** | **Non-assisted** |  |  |  |  |  |  |  |  |  |
|  | Senior vs AI system | **<0.0001** | **<0.0001** | **<0.0001** | **<0.0001** | 0.549 | **<0.0001** | **0.001** | **0.004** | 1.000 |
|  | Junior vs AI system | **<0.0001** | **<0.0001** | **0.013** | **<0.0001** | 0.118 | **<0.0001** | **<0.0001** | **<0.0001** | 0.625 |
|  | **AI-assisted** |  |  |  |  |  |  |  |  |  |
|  | Senior vs AI system | **0.001** | **<0.0001** | **<0.0001** | **<0.0001** | 1.000 | **<0.0001** | **0.006** | **0.003** | 0.375 |
|  | Junior vs AI system | **<0.0001** | 0.075 | **0.034** | **<0.0001** | 0.289 | **<0.0001** | **<0.0001** | **0.007** | 0.250 |
|  | **AI-assisted ability** |  |  |  |  |  |  |  |  |  |
|  | Senior | 0.071 | 0.500 | **0.016** | 0.070 | 0.344 | 0.121 | 0.267 | 0.607 | 0.500 |
|  | Junior | **<0.0001** | **<0.0001** | **<0.0001** | **0.001** | 0.629 | **<0.0001** | **0.012** | **0.011** | 1.000 |
| **Sensitivity** | **Non-assisted** |  |  |  |  |  |  |  |  |  |
|  | Senior vs AI system | - | **<0.0001** | 0.055 | **<0.0001** | 0.125 | - | **0.001** | 1.000 | 1.000 |
|  | Junior vs AI system | - | **<0.0001** | **<0.0001** | **<0.0001** | 0.125 | - | **<0.0001** | 0.219 | 1.000 |
|  | **AI-assisted** |  |  |  |  |  |  |  |  |  |
|  | Senior vs AI system | - | **<0.0001** | **0.003** | **<0.0001** | 0.250 | - | **0.039** | 0.125 | 1.000 |
|  | Junior vs AI system | - | **0.002** | **0.001** | 1.000 | 0.500 | - | **0.002** | 0.625 | 1.000 |
|  | **AI-assisted ability** |  |  |  |  |  |  |  |  |  |
|  | Senior | - | **0.003** | 0.085 | **0.013** | 1.000 | - | 0.109 | 0.125 | 1.000 |
|  | Junior | - | **<0.0001** | **0.001** | 0.481 | 0.500 | - | **0.013** | 0.625 | 1.000 |
| **Specificity** | **Non-assisted** |  |  |  |  |  |  |  |  |  |
|  | Senior vs AI system | - | **<0.0001** | **0.001** | **<0.0001** | 1.000 | - | 0.687 | **0.001** | 1.000 |
|  | Junior vs AI system | - | 0.872 | **<0.0001** | **<0.0001** | 1.000 | - | 0.250 | **<0.0001** | 0.625 |
|  | **AI-assisted** |  |  |  |  |  |  |  |  |  |
|  | Senior vs AI system | - | **<0.0001** | **<0.0001** | **<0.0001** | 1.000 | - | 0.250 | **0.039** | 1.000 |
|  | Junior vs AI system | - | 0.708 | 0.099 | **<0.0001** | 1.000 | - | 0.250 | **<0.0001** | 0.625 |
|  | **AI-assisted ability** |  |  |  |  |  |  |  |  |  |
|  | Senior | - | **<0.0001** | 0.126 | 0.736 | 0.180 | - | 1.000 | 0.065 | 0.500 |
|  | Junior | - | **<0.0001** | **0.001** | **<0.0001** | 0.302 | - | 1.000 | **0.019** | 1.000 |

IPCLs, intrapapillary capillary loops; AI, artificial intelligence; EP, epithelium; LPM, lamina propria; MM, muscularis mucosa; SM1, slight infiltration of the submucosa (< 200 μm); SM2, deeper infiltration of the submucosa($\geq$ 200 μm).

**
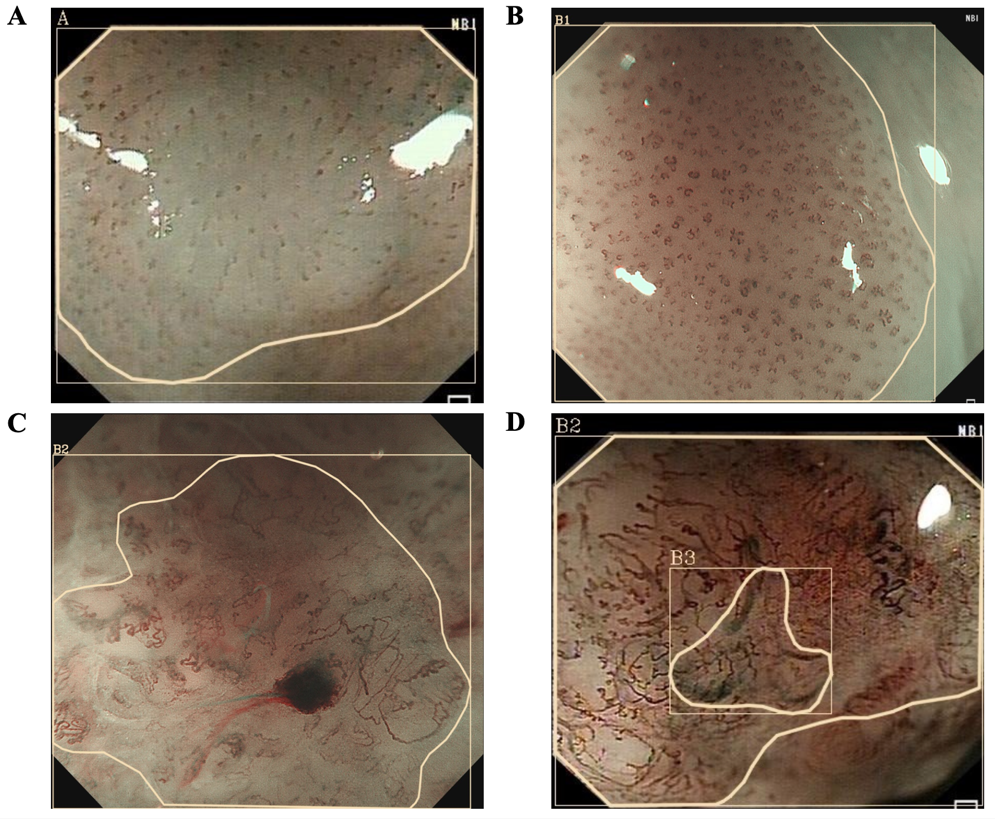
**

**Fig.S1** Examples of images annotated by experienced endoscopists according to IPCLs classification. IPCLs, intrapapillary capillary loops. (A) Type A vessels. (B) Type B1 vessels. (C) Type B2 vessels. (D) Type B2 and Type B3 vessels.


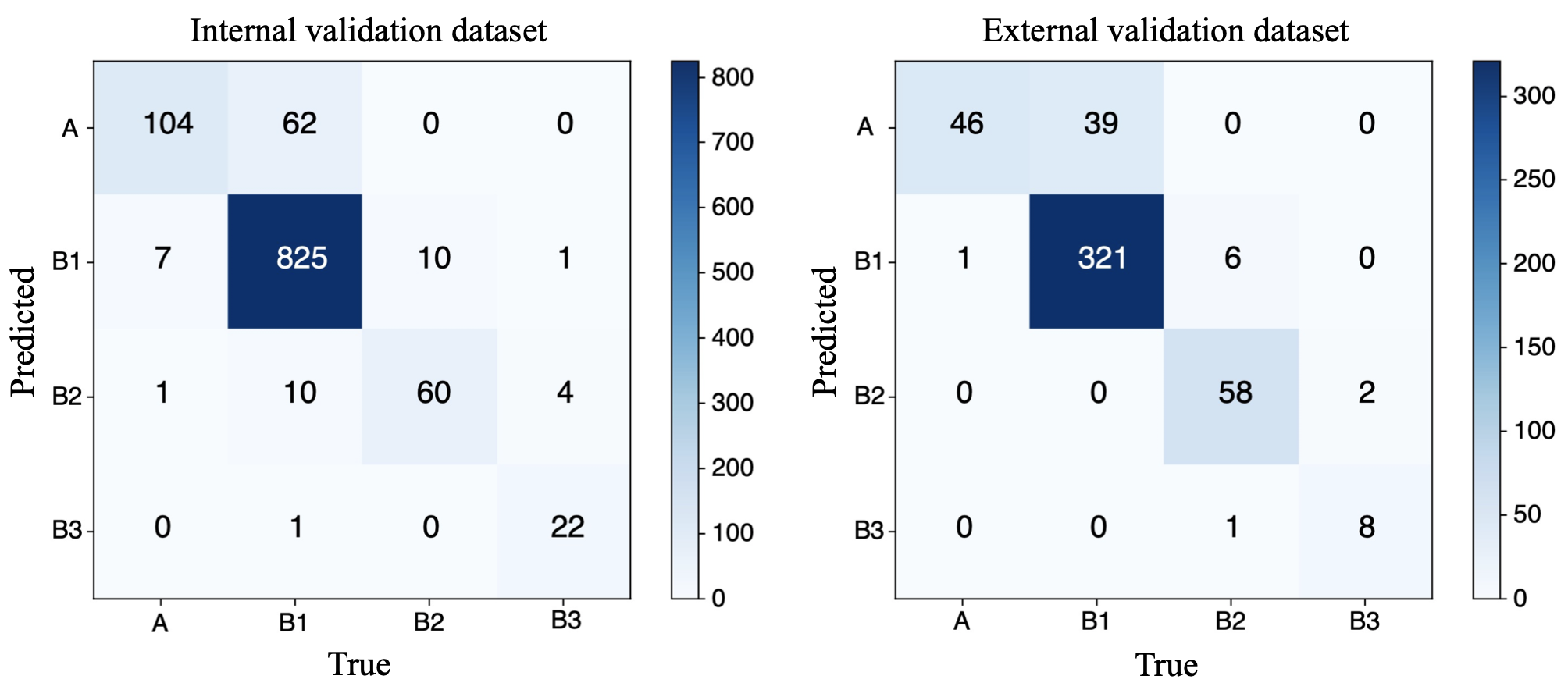


**Fig.S2** Performance of the artificial intelligence system in the internal and external validation datasets.

**
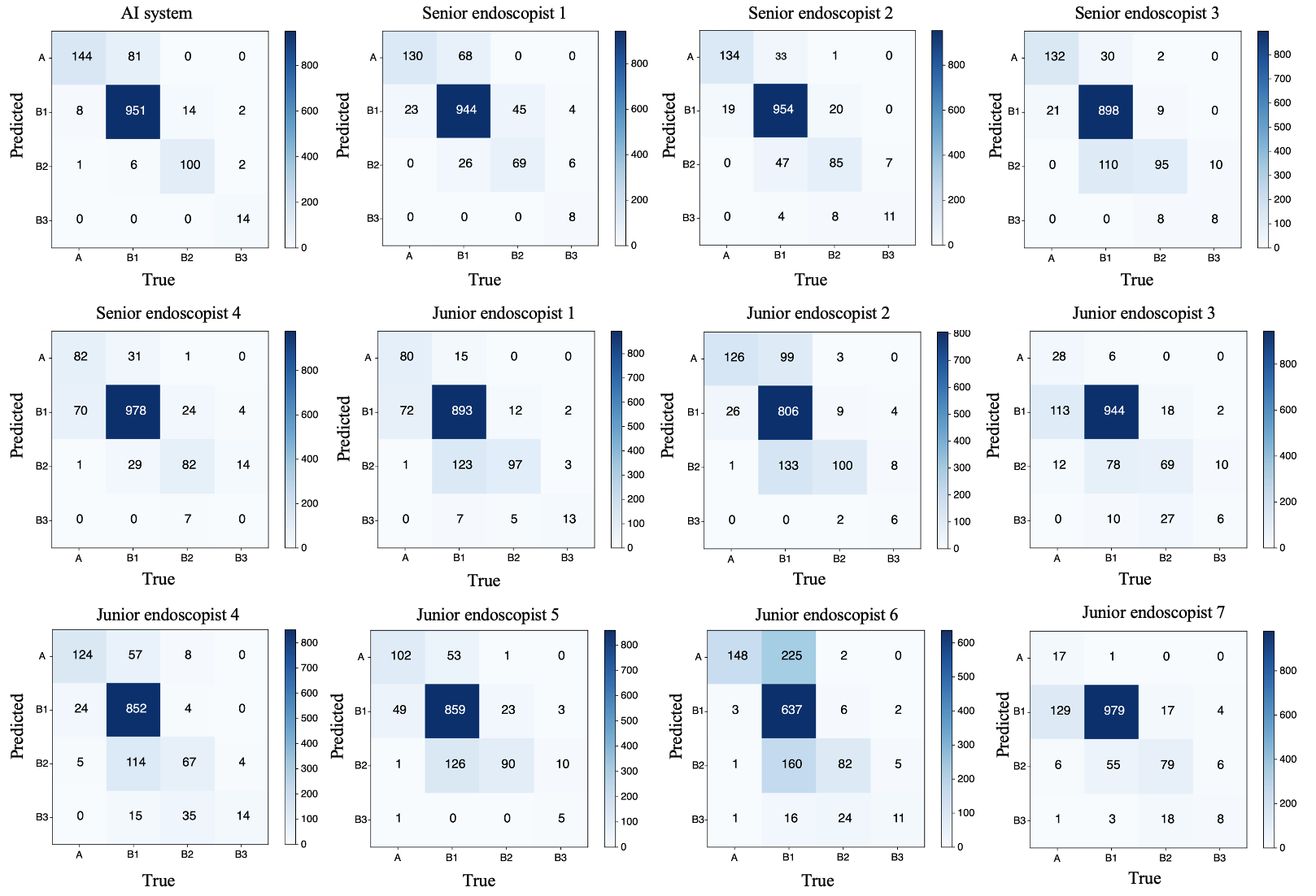
**

**Fig.S3** The diagnostic results of the AI system and eleven endoscopists without AI system assistance in the diagnosis of IPCLs subtypes in the ER validation dataset. AI, artificial intelligence system. IPCLs, intrapapillary capillary loops. ER, endoscopic resection.

**
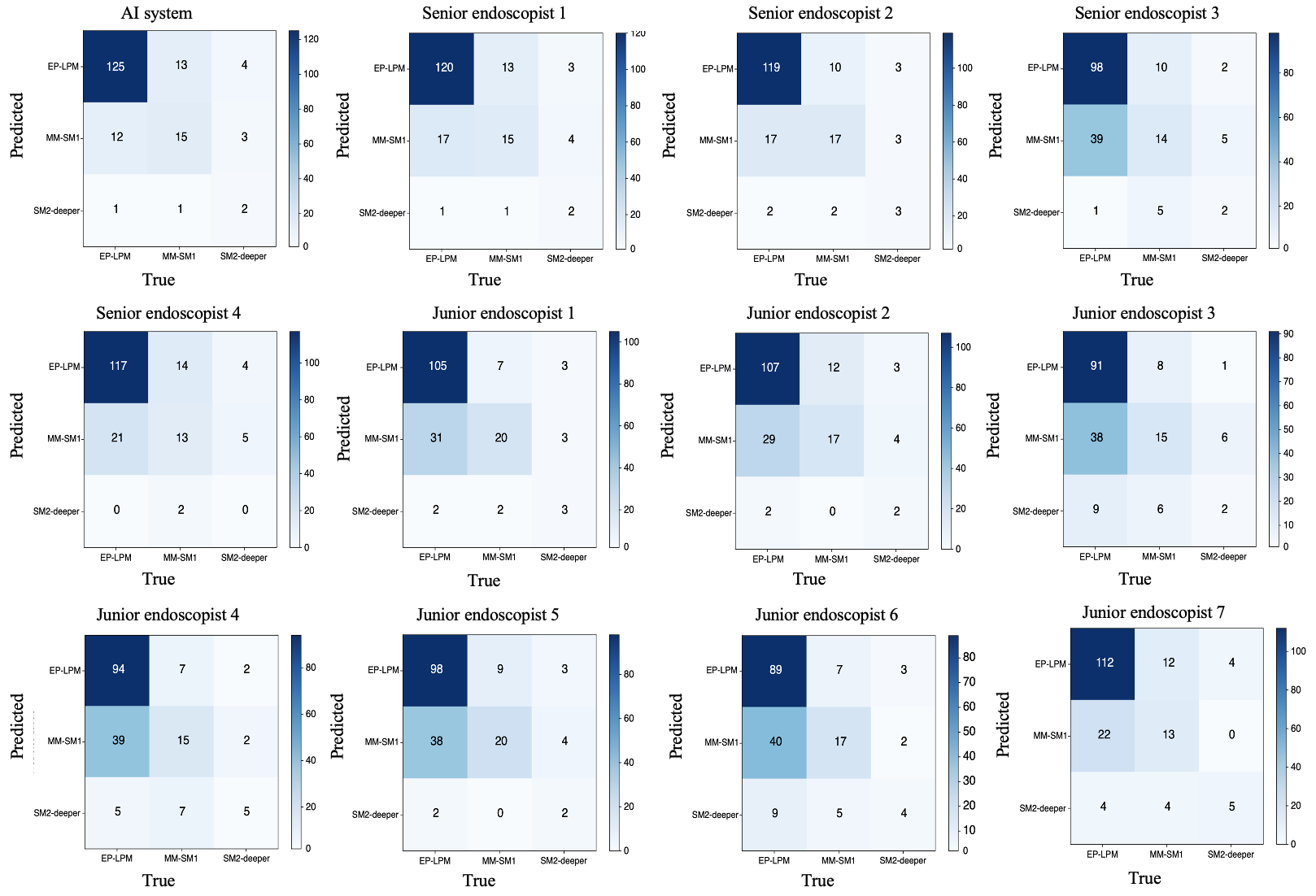
**

**Fig.S4** The diagnostic results of the AI system and eleven endoscopists without AI system assistance in the prediction of invasion depth in the ER validation dataset. AI, artificial intelligence system. IPCLs, intrapapillary capillary loops. ER, endoscopic resection.

**
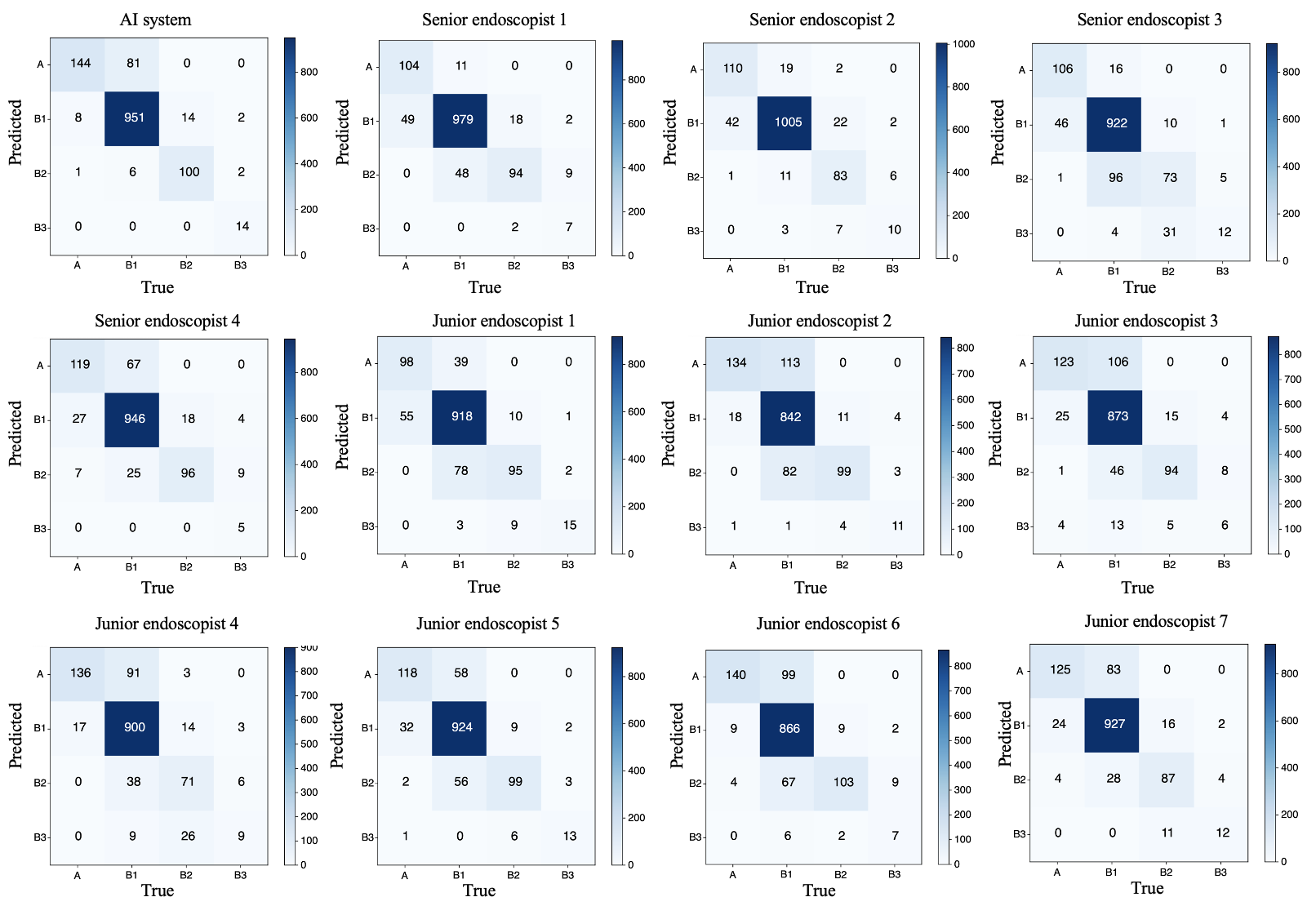
**

**Fig.S5** The diagnostic results of the AI system and eleven endoscopists with AI system assistance in the diagnosis of IPCLs subtypes in the ER validation dataset. AI, artificial intelligence system. IPCLs, intrapapillary capillary loops. ER, endoscopic resection.


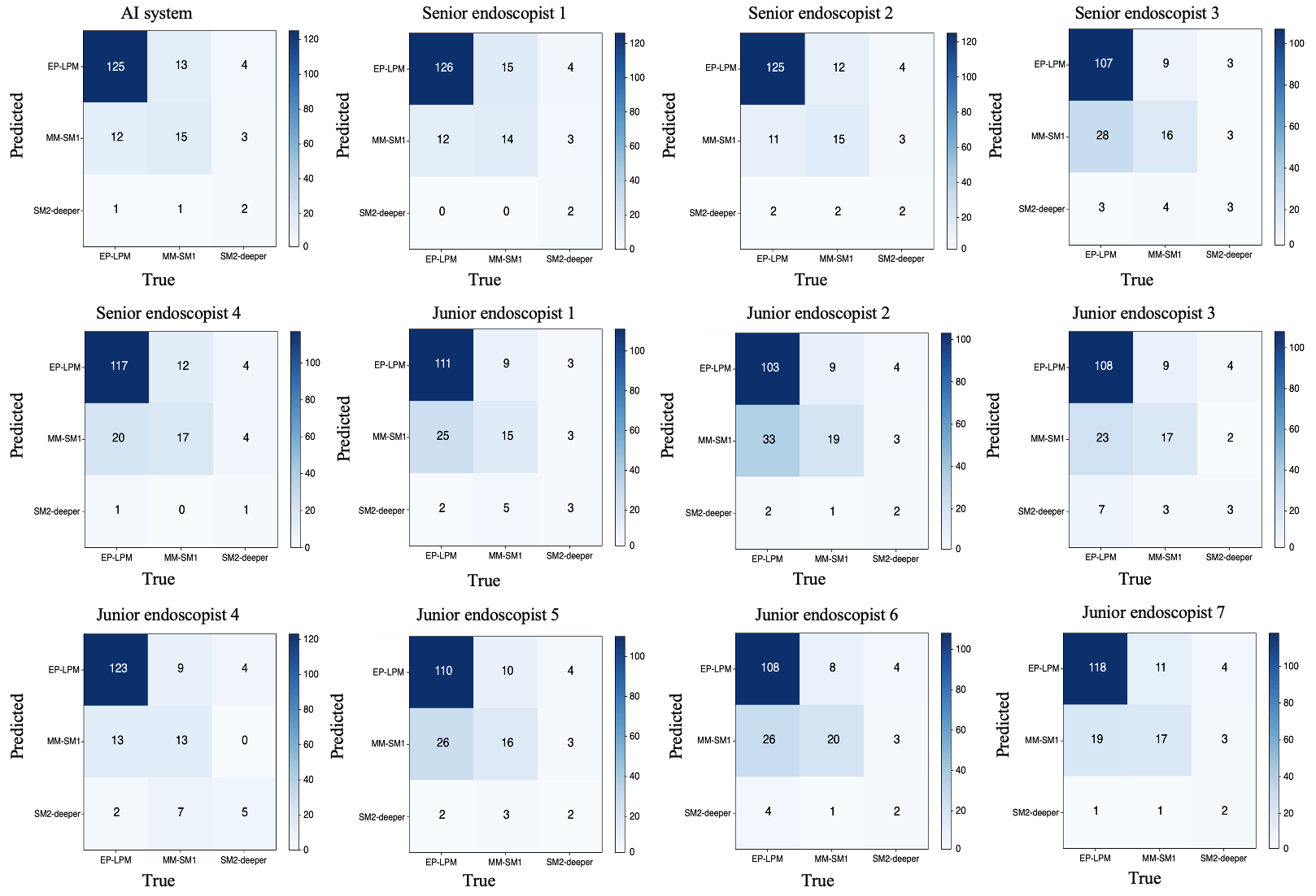


**Fig.S6** The diagnostic results of the AI system and eleven endoscopists with AI system assistance in the prediction of invasion depth in the ER validation dataset. AI, artificial intelligence system. IPCLs, intrapapillary capillary loops. ER, endoscopic resection.
